# FAP-specific re-directed T cells first in-man study in malignant pleural mesothelioma: experience of the first patient treated

**DOI:** 10.1186/2051-1426-3-S2-P120

**Published:** 2015-11-04

**Authors:** Magdalena Pircher, Petra Schuberth, Pratiksha Gulati, Simon Sulser, Walter Weder, Alessandra Curioni, Christoph Renner, Ulf Petrausch

**Affiliations:** 1 Department of Oncology, University Hospital Zurich, Zurich, Switzerland; 2 Department of Anesthesiology, University Hospital Zurich, Zurich, Switzerland; 3 Department of Thoracic Surgery, University Hospital Zurich, Zurich, Switzerland; 4 Department of Oncology, Private Clinic Hirslanden Zurich, Zurich, Switzerland

## Background

Median survival of advanced malignant pleural mesothelioma (MPM) is less than 2 years, and novel treatments are urgently needed. Immunotherapy with adoptive T cell transfer is an attractive approach that could be added to current therapeutic concepts. Fibroblast activation protein (FAP) is expressed by all subtypes of MPM [1] and can serve as target of re-directed T cells harboring an anti-FAP chimeric antigen receptor (CAR) leading to site-specific T cell activation.

## Patients and methods

This is a Phase I study with the primary clinical endpoint of safety and the secondary endpoints of feasibility and immune monitoring. Immune monitoring includes measurement of T cell subpopulations, cytokines and inflammatory parameters. This is the first *in human* application of FAP-specific autologous re-directed T cells in patients with newly diagnosed, inoperable MPM in the malignant pleural effusion. We report here on the first patient treated in February 2015. At that time, he was 74 years old and had received the diagnosis of MPM in November 2014. After 2 cycles of standard chemotherapy (Cisplatin/Pemetrexed), 250 ml of peripheral blood were collected (day -21), CD 8 T cells extracted and retro-virally transduced with a FAP-specific CAR, while the patient was receiving the 3^rd^ cycle of chemotherapy. On day 0 1x10^6^ CAR T cells were injected under sterile conditions into the pleural effusion through a pleural drainage on the intensive care unit (ICU), followed by 48 hours ICU surveillance. Simultaneously, cytokine levels were measured in the serum and in the pleural effusion.

## Results

In this first patient, we demonstrated safety and technical feasibility of the application of re-directed T cells. No adverse events due to the injection were observed, in particular no immune-mediated toxicity (e.g. cytokine storm, inflammation). *Ex vivo* analysis showed antigen-specific activity and function of re-directed T cells.

## Conclusion

Re-directed T cells against FAP could be a safe and feasible treatment option for patients with MPM in the future. Further studies are needed to demonstrate anti-tumor efficacy.
